# Relationship between Cognitive Function and Sway of Body in Standing Posture: A Cross-Sectional Study

**DOI:** 10.3390/geriatrics8020029

**Published:** 2023-02-25

**Authors:** Takao Naito, Yume Suzuki, Kotaro Yamasue, Kyoko Saito, Masanari Umemura, Narumi Kojima, Hunkyung Kim, Yosuke Osuka, Yoshihiro Ishikawa, Osamu Tochikubo

**Affiliations:** 1 Graduate School of Medicine, Yokohama City University, Yokohama 236-0004, Japan; 2 Department of General Internal Medicine, Yokohama City University Medical Center, Yokohama 232-0024, Japan; 3 Research Team for Promoting Independence and Mental Health, Tokyo Metropolitan Institute of Gerontology, Tokyo 173-0015, Japan

**Keywords:** cognitive function, MMSE, standing balance, sway of head, sway of foot pressure

## Abstract

Background: The influence of neurological or balance dysfunction on cognitive impairment has not been well studied. We compared the results of the balance test, measured by either head or foot sway to consider whole body sway, with those of the cognitive impairment test. Methods: Individuals of either gender, aged over 60 years, underwent a 30 s balance test. We measured sway while standing on one-leg or two-legs. Sway was evaluated by the distance or area of movement of the head or foot pressure. We also evaluated the effect of visual condition: eyes-open (EO) or -closed (EC). The Mini-Mental State Examination (MMSE) was used to evaluate the degree of cognitive impairment. Results: The head sway area standing on one leg was significantly correlated to MMSE score with EO (correlation r = −0.462). In standing on two legs, no sway test results showed a significant correlation to MMSE scores with EO. With EC, the magnitude of sway became greater, and was significantly correlated to MMSE scores in the head distance. Conclusion: Although the correlation between head sway and MMSE was not strong, head sway showed a stronger correlation than did foot pressure sway. Standing on one leg, as measured by head sway area, may thus predict cognitive impairment.

## 1. Introduction

Cognitive dysfunction or dementia is a major problem in many aging countries in US, EU, and Asia. However, in the early stage of cognitive dysfunction, it is difficult to determine its etiology [1,2]. Cognitive dysfunction is often accompanied by motor dysfunction, such as gait or fall, as well as perceptual and memory dysfunctions [3,4,5,6,7]. Thereby, detecting the development of cognitive dysfunction in the early stage is particularly important. Currently, the Mini-Mental State Examination (MMSE), in which verbal questions related to cognitive function, are questioned, is widely used to evaluate cognitive impairment in medical facilities. The MMSE is subjective, as it is readily influenced by an individual’s condition on the day of assessment. However, the MMSE is the most broadly used in the world. It has been shown to have a high sensitivity of 81% and specificity of 89% for detecting dementia. Therefore, the MMSE was considered sufficient for this evaluation criterion and was applied [8,9]. However, for detecting dementia, the use of objective and quantitative analytical methods that are accessible is necessary.

A candidate for the objective evaluation of cognitive impairment is the standing postural balance. However, the influence of balance dysfunction on cognitive impairment has not been well studied. Previous studies have reported a correlation between standing posture balance and cognitive dysfunction [10]. The evaluation methods for postural balance include the Mini-BESTest for dynamic balance [11], the foot pressure test for static balance [12,13], or the Romberg test [14]. The Romberg test compares between sway and standing time with eyes-open (EO) or eyes-closed (EC), while the individual is standing on two legs or one leg. The ability to maintain standing balance depends on neural signals of visual information, somatic sensations (from the muscle spindles of the legs), and vestibular sensations indicating gravity transmitted to the cerebellum and brainstem via nerves. Based on these signals, the muscle spindle receives feedback to control balance. Therefore, standing balance is related to cognitive function that attempts to maintain balance throughout the body. A previous study reported that one-leg standing time was related to the degree of cognitive impairment [15]. However, the head is an important element of the biokinetic chain. The head extends along the body’s midline, its location has been shown to have a significant impact on various human functions [16].

In the current study, we examined the usefulness of the head sway test compared with the conventional foot sway test to consider whole body sway, as the magnitude of sway must be greater in the head than in the foot. The head and foot pressure sway were measured during the two-legged and one-legged standing posture conditions. Moreover, the relationship with cognitive function was determined using the MMSE.

## 2. Materials and Methods

### 2.1. Participants

The participants were selected from neurologists at the University Hospital and older adults undergoing physical examinations to select individuals with cognitive decline. The sample size was targeted to be at least 100 participants. This study was approved by the Ethics Committee of the University (Yokohama City University: No. B170100016). The participants provided oral informed consent prior to enrolment in the study. All participants in this study were aged over 60. When interviewed, we excluded individuals with visual impairment (blindness), wheelchair users, as well as individuals requiring support to walk or with severe pain in the knees or back.

### 2.2. Sway Measurement Equipment

**Figure 1:**
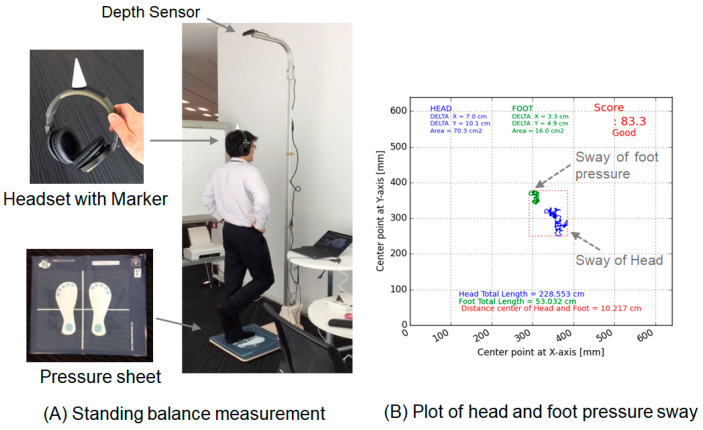
Head and foot pressure sway test. (**A**) Wearing a headset with a marker, the participant stood on the footprint for 30 s. Head and foot sway was measured with his/her eyes open or closed. A representative result of the one-leg standing test is shown. (**B**) Visualization of head and foot pressure sway by plotting is shown.

To measure body balance, both head and foot pressure sways were measured. A depth sensor (Intel RealSense D415, Sampling frequency: 9 fps) placed overhead was used to ensure the detection of sway at the center of the head (Figure 1A). Sway of the center of foot pressure was measured using a pressure mat (Sumitomo-Riko SR Soft Vision, Sampling frequency: 5 fps). On the pressure mat, a footprint marker indicated the place the participant was expected to stand. The participants followed instructions from a voice-over guide on when to start the measurement. In addition, they were instructed to stare at the marker positioned at eye level for 30 s during measurement. We measured sway distance (one-dimensional change) and area of sway (two-dimensional change). The sway of head or of foot pressure (foot gravity center) was independently measured.

Head sway and foot pressure sway were measured simultaneously during 30 s of standing. These sway measurements were evaluated based on the total moving distance and area of the moving trajectory (Figure 1B).

### 2.3. Cognitive Assessment Protocol

All participants underwent interviews to assess their cognitive function using the MMSE. MMSE interviews and scoring were conducted by medical physicians or supporters trained in MMSE interviewing. The MMSE had a total score of 30 points: the higher the score was, the higher the cognitive function was [9,17,18,19]. The MMSE comprised the following 11 subscale questions: orientation to time, orientation to place, immediate memory, serial seven calculation, delayed memory, naming, repetition, oral instruction, writing instruction, spontaneous writing, and copy a design.

### 2.4. Sway Test Protocol

We confirmed their basic attributes based on the information on the self-made questionnaire. The basic attributes were age, body mass index (BMI), fatigue on the day of assessment, exercise habits, sleep quality on the night before assessment, the influence of drinking, and presence/absence of pain in the lower back/knee. Grip strength, which is correlated with lower limb muscle strength, was measured to assess muscle strength [20]. Sleep quality was selected from a numerical value on a 4-point scale: good, normal, a little sleepy, and not at all sleepy, and fatigue on the day was on a 3-point scale: no, a little, and a lot on the questionnaire.

Head sway and foot pressure sway were then measured. The participants were evaluated for 30 s each, in the EO condition in the two-legs standing position, in the EC condition in the two-legs standing position, and in the EO condition in the one-leg standing position. We performed the sway test with EC, ablating visual correction and thus increasing the magnitude of sway independently from muscle strength.

### 2.5. Data Analysis

Mean, maximum, minimum, and standard deviation (SD) were calculated to conduct a demographic analysis of individuals and to test for equality of variances between groups. Sway during standing on two legs in the EO or EC conditions was evaluated. To verify the influence of visual information, sway difference (Δ) between the EO and EC conditions was added to the evaluation, because standing balance is integrated with sensory information from the somatosensory, vestibular, and visual systems [21]. The reason for evaluating the difference (Δ) was that the influence of the visual system was eliminated. Furthermore, the cognitive functions of the somatosensory and vestibular systems could be evaluated. This evaluation was based on the difference in the total moving distance of head sway (Δhl) and the difference of sway in the moving trajectory area (Δha) (Equations (1) and (2)). The sway of the center of foot pressure was also the difference between the total moving distance (Δfl) and the difference of the area of the moving trajectory (Δfa).

Δhl = EC_head_length-EO_head_length(1)

Δha = EC_head_area-EO_head_area(2)

Similarly, one-leg standing was evaluated during the EO condition only, and the evaluation parameters were hl (EO, the total moving distance of head sway), ha (EO, the moving trajectory area of head sway), fl (EO, the total moving distance of foot sway), and fa (EO, the moving trajectory area of foot sway).

Statistical analysis between sways (head and foot pressure) and MMSE subscale items (11 items including place, time, memory, language, and writing) were analyzed by the partial correlation coefficient. Adjustment variables were age, body mass index (BMI), fatigue on the day of assessment, sleep quality on the night before assessment, and grip strength. Sex was excluded from the variables because it was found to be correlated with grip strength.

## 3. Results

### 3.1. The Participant Characteristics

In total, 103 individuals (10 males and 93 females), as described in detail in Table 1, were recruited and were subject to the MMSE. Among the enrolled individuals who could stand on two legs, 54 individuals could stand on one-leg, and were subject to a sway test standing on one-leg with eye open (EO) condition. There was no significant difference in characteristics between the two groups: 103 versus 54. Standing on one leg with EC was too difficult to perform in most individuals.

### 3.2. MMSE Scores and Sway Test Standing on One-Leg

**Figure 2:**
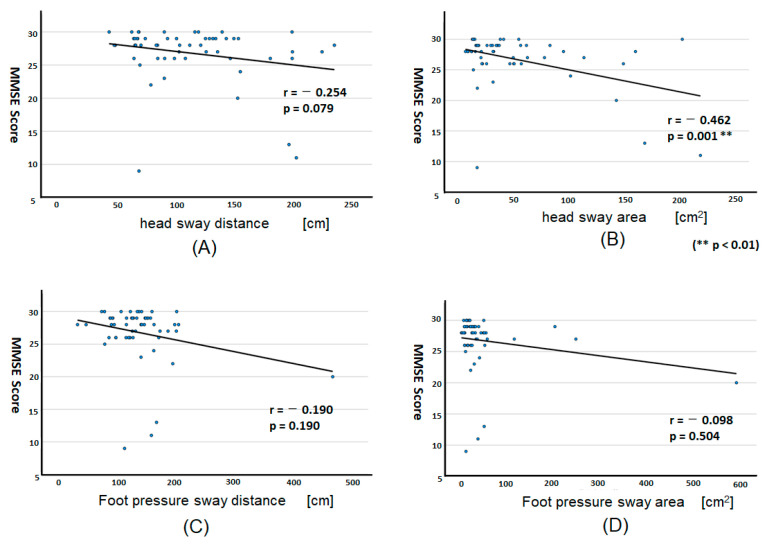
Correlation plotting between MMSE score and sway test results standing on one leg. Sway tests were performed when individuals were standing on one leg with EO (*n* = 54). The correlation was plotted for the head sway distance (**A**) and area (**B**), the foot pressure sway distance (**C**), and area (**D**). ** *p* <0.01.

We evaluated the usefulness of the sway test standing on one-leg. Only 54 individuals were able to stand on one-leg. A representative result is shown in Figure 2 In both distance and area, the magnitude of the head sway was greater than that of the foot, most likely because the head is more distant from the ground.

The relationship of the sway test results (sway distance and area) with MMSE scores was evaluated by using correlation coefficient analysis (Figure 2A–D). We found a statistically significant negative correlation (r = −0.462, *p* = 0.001) between MMSE scores and the head sway area, suggesting that the head sway area was larger with cognitive impairment (Figure 2B). This correlation coefficient of head sway (r = −0.254 for distance and −0.462 for area) was much better than that of foot pressure sway (−0.190 and −0.098, respectively), suggesting that the head sway was more useful than the foot sway to evaluate cognitive impairment.

We further examined specific parameters within MMSE, which included 11 subscales (Table 2). The relationship of the sway test results (sway distance and area) with each specific parameter of MMSE was evaluated. We found that the head sway area showed a significant correlation with 7 parameters while the head sways distance with 4 parameters. The foot pressure sway area showed a significant correlation with 2 parameters and that of distance with only one.

Putting together, our results suggest that the head sway test, in particular, as measured by sway area, was more useful than foot sway tests when evaluated during standing on one leg.

### 3.3. MMSE Scores and Sway Test Standing on Two-Legs

We then performed similar tests standing on two legs, which were readily performed by all individuals (*n* = 103). When they stood on two-legs with EO, the magnitude of sway per se became much less, as expected, and they did not show any significant correlation with MMSE scores. The magnitude of sway became greater with EC on two legs and showed a significant correlation with MMSE scores regarding head sway distance (correlation r = −0.353, *p* < 0.001) and area (correlation r = −0.228, *p* = 0.025). However, no significant correlation was found regarding foot pressure.

### 3.4. Sway Test Standing in the Difference between EO and EC

**Figure 3:**
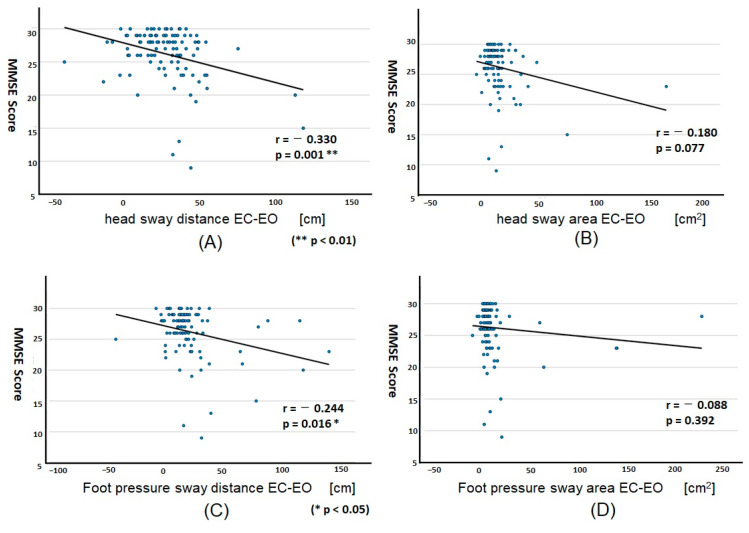
Correlation plotting between MMSE score and sway test results standing on two legs by EC-EO. Sway tests were performed when individuals were standing on two legs in EC and EO (*n* = 103), and the difference between EC and EO was obtained. The correlation was plotted for the head sway distance (**A**) and area (**B**), the foot pressure sway distance (**C**) and area (**D**). Note that a significant correlation was found in the head (**A**) and foot pressure sway (**C**).

We then used the difference (Δ) between EO and EC of the head or foot pressure sway test standing on two legs. (Figure 3).

The sway distance, both head (*p* = 0.001) and foot (*p* = 0.016), showed a significant correlation with MMSE score (Figure 3A,C). This correlation coefficient of head sway (r = −0.330 for distance and −0.180 for area) was much better than that of foot pressure sway (−0.244 and −0.088, respectively), suggesting that the head sway was more useful than the foot sway to evaluate cognitive impairment.

## 4. Discussion

We have evaluated the degree of cognitive impairment using MMSE scores [17,18,19] and evaluated their relationship with sway. The results showed that the two- and one-legged sway tests were significantly correlated with the degree of cognitive impairment evaluated by the MMSE. In the present study, head sway was newly measured in addition to conventional foot pressure sway. The correlation coefficient of head sway was higher than that of conventional foot pressure sway (Figure 2), which may affect the relationship with cognitive impairment. When the MMSE scores were classified into two subgroups (healthy or features of cognitive dysfunction) with a cutoff of 27/28, and the sway was compared, significant differences were obtained in head sway (area) for both EO and EC standing on two legs (*p* = 0.040 and *p* < 0.020, respectively). On the other hand, no significance was obtained for foot pressure sway, suggesting that head sway is related to cognitive dysfunction. In addition, postural sway requires attention. Since fatigue and sleep quality were possible influences on attention, these were used as adjustment variables and reflected partial correlations.

A multiple regression analysis was validated using the MMSE score as the dependent variable. The independent variable was tested in the case using the sway area of the head during one-leg standing with EO, which is relatively correlated with the MMSE score. Other independent variables added were age, BMI, fatigue during the day, sleep quality at the night, and grip strength. The results showed that the multiple regression equation was significant F = 0.004 (*p* < 0.01), but not highly accurate, as the adjusted R2 = 0.238 (Equation (3)). In the future, we would like to improve the accuracy by using a model that includes lower limb muscle strength.

MMSE = −0.04 × ha − 0.18 × ag + 0.24 × B + 0.91 × ftg +0.38 × slp − 0.09 × hg + 38.1(3)

(ha: area of head sway, ag: age, B: BMI, ftg: fatigue on the day, slp: sleep quality on the night, hg: grip strength)

A correlation existing between cognitive function and standing balance function has been revealed [10]. Moreover, it has been reported that the risk of falls increased with cognitive decline [3,6]. The ability to maintain standing balance depends on neural signals transmitted to the brain from visual information and somatic sensations. Therefore, it is reasonable to suggest that standing balance might be related to cognitive function, and that the head sway test could be sensitive when the entire body attempts to maintain balance.

Eye tracking [22], changes in physical movements (walking speed, stride length, and standing balance sway) [23,24], and brain imaging are currently used to detect early cognitive impairment in an objective manner. These tests are also valuable because they can determine whether imbalance during standing could be a result of brain-related cognitive impairment or weakened lower limb muscle strength. Although one-leg standing during the Romberg test may be able to verify the presence of locomotive syndrome for fall prevention, the posture places a heavy physical load on older adults. In addition, moderate exercise is required to test the correlation between body posture and cognitive impairment using dynamic balance tests, such as walking [11]. Thus, the above tests are valuable, but not very easy to perform in older patients. Static standing balance measurement may be a simple method that requires less physical load in older adults [25]. Swaying only during two-leg standing could screen for a large or small risk of cognitive decline.

Although testing with EO in the two-leg standing position was not significantly related to cognitive function when independently measured, it became significant when measured by the difference between EO and EC sway test (Figure 3). This supports our hypothesis that evaluating the difference in sway between the EO and EC conditions could eliminate the influence of the visual system and evaluate the effect of cognitive decline in the somatosensory and vestibular systems.

The difference in foot pressure sway was not significant in the one-leg standing position. However, head sway was significant (Figure 2). This may be because the center of foot pressure of one leg moved only within the area of the footprint, and consequently, the range of sway became smaller. Head sway, in contrast, detects the sway of the entire body, making it easy to monitor the sway per se. The correlation between MMSE scores and standing sway was also higher in head sway than in foot pressure sway. Another explanation for this difference may include, with declining cognitive function, postural correction response is also delayed and thus, leading to increased head sway [10].

Finally, our study was limited by the small number of participants. In addition, individuals who could not stand on two legs were excluded from the study. However, given the relationship between sway and cognitive function, the findings of sway in the standing position might have the potential to evaluate both the physical and cognitive status. Since balance could be improved by practice, even in individuals with dementia [26], monitoring standing sway may be used to evaluate the effect of rehabilitation on improving cognitive function.

## 5. Conclusions

Although the correlation between head sway and MMSE scores was not strong, the head sway showed a higher correlation than did foot pressure sway considering the assessment for sleep quality and fatigue before the sway test. Standing on one leg, as measured by head sway area, may thus predict cognitive impairment. For those who could not stand on one leg, the head sway test standing on two legs with EC may be useful.

## Figures and Tables

**Table 1:** Characteristics of individuals who underwent the two-legs or the one-leg standing tests.

	Two-Legs (*n* = 103) Mean ± SD	One-Leg (*n* = 54) Mean ± SD	*p* Value
Age	77.52 ± 6.90	75.4 ± 6.11	0.057
Sex (male = 0/female = 1)	0.9 ± 0.30	0.93 ± 0.26	0.610
BMI	22.54 ± 3.32	21.9 ± 3.44	0.258
Grip strength	23.27 ± 6.33	24.46 ± 5.77	0.260
Fatigue on the day of assessment	0.57 ± 0.67	0.53 ± 0.69	0.686
Sleep quality	1.64 ± 0.71	1.78 ± 0.76	0.249
MMSE score	26.22 ± 3.96	26.8 ± 4.37	0.402

**Table 2:** Relationship between MMSE subscale and the sway test result in one-leg sway test (EO).

MMSE Subscale	Partial Correlation Coefficient							
	Head Sway				Foot Pressure Sway			
	Distance	*p* Value	Area	*p* Value	Distance	*p* Value	Area	*p* Value
Orientation to time	−0.242	0.093	−0.464 **	0.001	−0.148	0.309	−0.050	0.732
Orientation to place	−0.267	0.064	−0.509 **	0.001	−0.384 **	0.006	−0.295 *	0.039
Immediate memory	0.170	0.242	0.124	0.396	−0.109	0.457	0.052	0.723
Serial sevens	−0.289 *	0.044	−0.438 **	0.002	−0.085	0.559	−0.143	0.326
Delayed memory	−0.201	0.165	−0.295 *	0.040	−0.205	0.159	−0.098	0.504
Naming	−0.341 *	0.016	−0.492 **	0.001	−0.101	0.491	−0.033	0.820
Repetition	−0.171	0.239	−0.335 *	0.019	−0.076	0.602	0.113	0.438
Oral instructions	0.129	0.378	0.067	0.648	0.120	0.411	0.209	0.149
Writing instructions	−0.341 *	0.016	−0.492 **	0.001	−0.101	0.491	−0.033	0.820
Spontaneous writing	0.072	0.621	0.035	0.814	0.095	0.515	0.128	0.382
Copy a design	−0.317 *	0.027	−0.179	0.218	−0.080	0.585	−0.330 *	0.021

Covariates: age, BMI, fatigue on the day of assessment, sleep quality on the night before the assessment, and grip strength. * *p* < 0.05, ** *p* < 0.01.

## Data Availability

The data used to support the findings of this study are available from the corresponding author upon request. The data are not publicly available because they contain information that can compromise the privacy of research participants.
